# Nano-and Micromotors Designed for Cancer Therapy

**DOI:** 10.3390/molecules24183410

**Published:** 2019-09-19

**Authors:** Luisa Sonntag, Juliane Simmchen, Veronika Magdanz

**Affiliations:** 1 Chair of Physical Chemistry, TU Dresden, 01062 Dresden, Germany; luisa.sonntag@chemie.tu-dresden.de; 2 Chair of Applied Zoology, TU Dresden, 01062 Dresden, Germany; Veronika.magdanz@tu-dresden.de

**Keywords:** nano- and micromotors, cancer, drug delivery, loading and release, challenges

## Abstract

Research on nano- and micromotors has evolved into a frequently cited research area with innovative technology envisioned for one of current humanities’ most deadly problems: cancer. The development of cancer targeting drug delivery strategies involving nano-and micromotors has been a vibrant field of study over the past few years. This review aims at categorizing recent significant results, classifying them according to the employed propulsion mechanisms starting from chemically driven micromotors, to field driven and biohybrid approaches. In concluding remarks of section 2, we give an insight into shape changing micromotors that are envisioned to have a significant contribution. Finally, we critically discuss which important aspects still have to be addressed and which challenges still lie ahead of us.

## 1. Introduction

Nano- and micromotors are small-scale devices, capable of transforming energy efficiently into motion [1,2]. Since on the small scale on-board fuel transport is limited, these devices need to convert the energy from the environment, which requires the presence of energetic fields or chemical fuels. In nature, motile microorganisms are the evolution’s solution to this problem [3,4,5,6], and they have developed efficient mechanisms to propel. Inspired by that, for almost two decades scientists have proposed various actuators on the microscale that can not only move, but increasingly solve complex tasks.

The famous science-fiction movie “The fantastic voyage” from 1966 illustrated the possible advantages of miniaturized medical intervention, such as more precise transportation of drugs, higher penetration capabilities and non-invasive operation. However, the strategy they employed was shrinking down a macroscale submarine, which would collide with the different physical constraints imposed by different Reynolds numbers governing the motion (for more detailed discussion see references [7,8,9]).

In recent years, scientists are working on putting this vision into reality and look intensively at the possibility to use active motion for drug delivery. Since there is a range of excellent reviews available on this broad topic [10,11,12,13,14,15,16,17,18,19], in this manuscript we will strictly focus on the envisioned use of nano- and micromotors in cancer therapy, mainly the delivery of cancer related medication, even though there has also been significant progress on surgery related microtools [20,21]. Despite the fact that most of the earlier motors were made of rigid, inorganic materials [22,23] and rely on the decomposition of hydrogen peroxide, the field has grown up and now a wide range of soft [24,25] and composite materials have been introduced. These combinations do not only allow the exploration of different new fuels and propulsion strategies, but also a broad range of new, biomedically relevant applications [16]. Further applications of nano- and micromotors range from environmental remediation [26,27] to sensing tasks [28,29,30] depending on the specific design of the swimming device.

Additionally, many different applications in the field are suggested to target various types of diseases. One example is the slow breakdown of blood coagulations or acidic propulsion of Mg particles in the mouse stomach for acid neutralization [31] and drug delivery in the digestive system [32,33,34]. All these have been explained recently in different reviews on the topic [10,35,36,37,38].

The review is structured in a way that we explain the different propulsion mechanisms categorized into chemical, external energy and biohybrid approaches. The chemical nano-and micromotors are discussed according to their fuel, composition and morphology. We describe electric, magnetic, acoustic, optical and thermal actuation methods in the section of external energy driven nano- and micromotors. Within the biohybrid nano- and micromotors, we distinguish between biological components serving as structural, loading or propulsion units. Readers familiar with the micromotor related research might find Section 3 and Section 4 insightful regarding current challenges in terms of cancer targeting nano- and micromotors, as well as adjacent fields (imaging, release and guidance strategies).

The advantages associated with the ideal case of a fictional drug delivering nano- and micromotor lie at hand:

- Reduced drug concentration due to specific targeting possibilities, which results in less side effects and in lower costs.
- A reduced amount of active pharmaceuticals consequentially leads to a reduced amount of waste products. Generally, pharmaceuticals are metabolized or simply excreted by the body after their use, leading to high concentrations in sewage waters, which are very often difficult to remediate in water treatment plants. This is not only the case for cancer medicine, but due to the high cytotoxicity of these compounds are especially dangerous for living organisms in aquatic systems [39].
- Compared to passive drug delivery agents, the use of nano- and micromotors bears the advantage, that propulsion is independent from the blood flow. Smart engineered drug carriers display additional advantages, especially when combined with other approaches, such as encapsulation, targeting moieties on the surface, cell or gene delivery. Drug delivery with on-demand site-specific release becomes especially useful for pharmaceuticals causing severe side effects, such as those used in cancer therapy.
- The high variability among nano-and micromotors allows the incorporation of different features provided by the drug delivery community, such as drug protection by encapsulation [40,41,42], selectivity by combination with selective biomarkers and, in case of nano-scaled motors, the ability to penetrate tissue.
- A remote trigger mechanism to release the drug at the desired location can be implemented using micromotors. This has been introduced into micromotion using piezo induced changes [43] but also in strategies like molecular valves are envisioned [44,45]. The full overview of demonstrated examples of controlled drug release for cancer therapy by nano-and micromotors is displayed in Table 1, Table 2 and Table 3.

Apart from the directly related advantages, the environmental benefits of targeted drug delivery are a globally strong argument for putting efforts into the development of nano- and micromotors. The problem concerning drug residues in waste water is growing and carried into crops, food, and also other sources such as abuse of pharmaceuticals. However, many of the ideas seem rather science fiction than close to being implemented. It is easy to list opposing factors that still inhibit the implementation of active motion in drug delivery:

- The first point to list here is often (also in many current publications) the use of highly reactive fuels, such as hydrogen peroxide or hydrazine or ultraviolet (UV) light to achieve propulsion. Even though alternative strategies are being developed, many propulsion mechanisms are not fully compatible with the use in body fluids, but this will be discussed in detail in Section 2.
- Particles require nanoscale dimensions for passage through tissues. For achieving an enhanced permeability and retention (EPR) effect usually dimensions up to 180 nm are assumed. According to a study of the Fischer group for penetrating mucus a particle size not larger than 100 nm seems to be beneficial for passing through hydrogel networks [46]. Additionally, the particle size plays an important role in biologically relevant processes such as the circulation and biodistribution of nanoparticles. Currently, most balistically moving nano-and micromotors are in the micrometer scale rather than lower nanometer scale.
- As an opposing fact to the previous point, Brownian rotation dominates for particle sizes below 800 nm. Here, the particle orientations are randomized and only Brownian motion or random walks are expected. Motion control is, however, crucial for targeted drug delivery, but fabrication, integration, modification and motor-cargo integration are still rather challenging below 100 nm.
- The materials employed for nano- and micromotor design are mostly chosen for functionality rather than biocompatibility, which can often lead to toxic components in motors. For clinical applications these need to be adapted and optimized concerning their non-toxicity, as well as their ability to be scaled up to allow high through-put fabrication.
- Most nano- and micromotors are still rather slow, so that large distances in the body would require long action times or, if applicable, high fuel concentrations. Further, careful navigation to avoid tissue damage might still be recommendable or injection to areas close to the tumor site is necessary.

## 2. Propulsion Strategies

Researchers have developed several approaches for propelling nano- and micromotors. While most early micromotors clearly belonged to the phoretic driving mechanism, the desire to emulate the swimming strategy of living microorganisms and to avoid oxidative and toxic fuels, drove developments into many different directions. 

Phoresis is the motion of an object in a gradient of different species, as will be specified later [8]. To create a self-phoretic swimmer, the gradient is generally induced by the colloid itself, often by an asymmetrically occurring chemical reaction [47]. The propulsion relies on the formation of a local gradient, which interacts in a thin, locally confined layer with the surface of the colloid. Throughout the literature it was shown that the nature of the gradient can be created from different solutes [48,49], electric charges [50] or thermal energy (temperature) [51,52]. The processes cause locally confined interactions and are often simplified as ’phoretic slip velocity’, which is defined as a thin layer interaction.

Field-induced propulsion of nano- and micromotors is occasionally not considered self-propulsion, since technically the external field is the origin of propulsion. Different approaches range from optical or magnetic trapping to alternating current (AC) electromagnetic fields, leading to a large variety of effects. 

Body deformation and mechanical interaction are often employed for propulsion in biological systems [24,25]. Even though biophoretic propulsion was predicted as early as in the 1970s, no such swimmer has been found yet. Most biological microswimmers have been proposed for therapeutic use, which will be discussed in detail. Biohybrid micromotors [53,54,55,56] generally move through mechanical movements that cause hydrodynamic flows in the surrounding of the swimmer. A drag is created through these non-reciprocal movements that acts on the swimmer and causes a forward motion. The absence of chemical effects in the motion mechanism simplifies not only theoretical modelling and simulations of these systems, also the interactions with the environment are more straight forward.

Each mechanism will be briefly introduced, discussed and most significant examples and their potential for cancer treatment will be summarized.

### 2.1. Chemically Based Strategies

**Figure 1:**
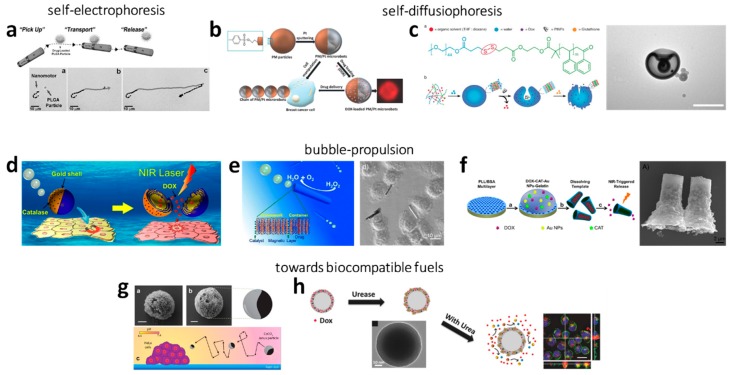
Summarizes selected examples of chemically driven nano- and micromotors: (**a**) Self-electrophoretic propelled Ni/(Au_50_/Ag_50_)/Ni/Pt nanowires picking-up and transporting polymer particles or liposomes loaded with DOX. Reprinted with permission of John Wiley and Sons, Kagan et al. [57], Copyright 2010. Self-diffusiophoresis in H_2_O_2_ of (**b**) DOX-loaded polymer/platinum microparticles, which could be magnetically guided towards breast cancer cells. Reprinted with permission of John Wiley and Son, Villa et al. [62], Copyright 2018. and (**c**) polymers in stomatocyte morphology loaded with platinum as catalyst and DOX as anticancer drug. The TEM images shows the stomatocytes, scale bar is 200 nm. Reprinted with permission from Tu et al. [61], Copyright 2017. (**d**) O_2_-bubble propelled robots upon reaction of H_2_O_2_ with catalase attached on DOX-loaded polymer/Au Janus particles. The drug could be released via NIR irradiation. Reprinted with permission from ref. [79]. Copyright 2014 American Chemical Society, (**e**) O_2_-bubble Pt/chinin/alginate nanorockets, Reprinted with permission of John Wiley and Sons, Wu et al. [89], Copyright 2013, and (**f**) Reaction of H_2_O_2_ and catalase on DOX- and Au-loaded microrockets, which released the drug upon NIR irradiation. Reprinted with permission from ref. [90]. Copyright 2015 American Chemical Society. (**g**) CO_2_-bubble propelled carbonate/Co Janus particles upon reaction with the acidic environment of HeLa cells. Reprinted with permission of Guix et al. [100], Copyright 2016. (**h**) Urea powered SiO_2_/urease nanoparticles that were loaded with DOX showing an improved effect on HeLa cells. Reprinted with permission of John Wiley and Sons, Hortelão et al. [70], Copyright 2017.

Phoretic propelled nano- and micromotors convert an external fuel into movement. These motors typically consist of at least two components: one inert material and one active material, e.g., a metal or an enzyme as catalyst. The motion is based on creating an asymmetric environment around the nano- and micromotors. This is either achievable by developing a concentration gradient and particle motion via self-phoresis or by producing bubbles (bubble propulsion) in which the thrust is caused by the recoil of bubbles. Hereby, commonly used fuels are H_2_O_2_, urea, glucose, water and acids or bases. The most extensively investigated fuel is H_2_O_2_ used for self-electrophoretic, self-diffusiophoretic or bubble-driven movement. An overview of chemically driven cancer-targeting nano-and micromotors can be found in Table 1 and selected examples are presented in Figure 1.

#### 2.1.1. Electrophoresis

First demonstrated by Sen’s group in 2004, Au-Pt nanorods moved in the presence of H_2_O_2_. Here, a proton gradient was generated by the decomposition of H_2_O_2_ to O_2_ and H^+^ on the Pt side and the generation of H_2_O from reaction of H_2_O_2_ with the H^+^ produced on the Au side. Both sides worked together as a short-circuited galvanic cell and the motion relied on self-electrophoresis [23,50]. In 2010, Wang’s group introduced Ni/(Au_50_/Ag_50_)/Ni/Pt nanowires for pick-up and transport of polymer particles or liposomes loaded with the anticancer drug doxorubicin (DOX). The metal nanowires were accelerated by self-electrophoresis in 5 wt% H_2_O_2_ [57].

#### 2.1.2. Self-Diffusiophoresis

##### H_2_O_2_ as Fuel

Another propulsion mechanism in H_2_O_2_ is self-diffusioresis, first introduced by Golestanian [47] and experimentally realized by Howse et al. [58]. The latter describes a motion caused by a self-generated local concentration gradient of the produced, dissolved O_2_. Wilson and co-workers designed copolymers in stomatocyte morphology and attached Pt nanoparticles inside their cavities [59,60]. The fuel H_2_O_2_ could enter the stomatocytes and decomposed at the Pt sites into O_2_ and H_2_O. Consequently, a rapid discharge was generated as well as a thrust, which induced directional movement. In this context, the group also presented thermo-responsive stomatocytes. At higher temperatures (40 °C) the stomatocyte opening was narrowed and the H_2_O_2_ supply was decreased, leading to a Brownian-like, decelerated movement [60]. Furthermore, DOX-loaded stomatocytes were presented (200 nm), which could chemotactically be guided towards human cervical cancer (HeLa) cells along a H_2_O_2_ gradient. Moreover, these particles were biodegradable [61]. Recently, Pumera and co-workers synthesized superparamagnetic microspheres (PM) out of of iron oxide microparticles (γ-Fe_2_O_3_) and covered these with a polymeric porous layer functionalized with sulphonyl esters (tosyl groups). The 4.3 µm sized Janus particles were derived by sputtering one half of the particles with Pt. These self-propelled micromachines moved due to a fluid flow generated by the catalytic decomposition of H_2_O_2_ on the Pt side. The particles were investigated as drug carriers. For this, DOX could be attached at the nanomotors’ surface by binding at the tosyl groups and magnetically guided towards breast cancer cells. A shrinkage of the cancer cells was observed already 10 minutes after interaction [62].

Unfortunately, high concentrated H_2_O_2_ is, due to its strong oxidation strength, incompatible in living organisms and limits practical biomedical application [63]. Otherwise, H_2_O_2_ is endogenously produced, e.g., in the mitochondria [64] or by activated cancer cells (0.2 nmol to 0.5 nmol/10^4^ cells/h) [65], and the human body modulates levels of H_2_O_2_ (among other reactive oxygen species) through antioxidant protective mechanisms [65]. The endogenous concentrations of H_2_O_2_ can even serve as fuel and for guidance [61,66]. The use of H_2_O_2_ as fuel is under debate as on the one hand, it is considered as being produced during carcinogenesis, but on the other hand it can support cancer cell death [67]. Nevertheless, the currently needed concentrations of hydrogen peroxide for active motion of catalytic nano- and micromotors is several orders higher than what is found in living tissue or produced by cells. 

##### Developments towards More Biocompatible Fuels

Hence, researchers aim for a propulsion under physiological conditions and environments as well as biocompatibility. In order to maintain the viability of the biologically targets, attempts have been made towards exploring biologically available fluids/substances as fuels. Good candidates are urea or glucose as they exist in biological environments with concentrations of 5–10 × 10^−3^ M [68,69]. The mentioned nano- and micromotors contain enzymes (catalase (Cat), urease, or glucose oxidase (GOx)) and the reactions evolve in principle as follows:

Urea + 3 H_2_O → CO_2_ + 2 NH_4_OH, (1)

also described are:

(NH_2_)_2_CO + H_2_O → CO_2_ + 2NH_3_ [70], (2)

d-Glucose + H_2_O +O_2_ → d-gluconic acid + H_2_O_2_, (3)

Attempts have been made to investigate urea as fuel for particle propulsion [70,71,72,73], but drug loading and in vitro experiments are rare. Hortelão et al. attached urease on mesoporous silica nanoparticles (350 nm) and presented their self-propulsion in ionic media upon reaction with urea. The study further showed that the nanomotors, in comparison to passive carriers, show an enhanced effect on cancer cells [70].

Very recently Sanchez’ group presented a silica based urease driven nanomotor (480 nm), that was able to perfom enhanced diffusion in rat urine. This nanomotor was functionalized with polyethylene glycol (PEG) and an anti fibroblast growth factor, so that it binds to the fibroblast in cancer speroids and inhibits the proliferation [74].

Another urease-driven Janus nanomotor was recently proposed for drug delivery in tumours. In this case, camptothecin was loaded into mesoporous silica nanoparticles and covered on one side with urease for propulsion in the tumour environment that contains urea and hyaluronic acid on the other side for improved penetration into the tumour and enhanced cellular uptake. Additionally, these Janus nanomotors are loaded into fiber fragments for intratumoral administration to achieve a high retention and gradual release of the nanomotors [75].

Furthermore, glucose is under investigation as fuel for actuating nano- and micromotors [71,76,77,78]. Since the presented studies lack cell experiments, in the following, the different concepts are presented shortly. Wilson and co-workers immobilized enzymes inside the previously mentioned stomatocyte particles, which were propelled by O_2_ generated from the reaction with glucose [77]. Ma et al. presented hollow mesoporous silica Janus nanorobots (390 nm) which either work with urea or glucose (or even H_2_O_2_) by immobilizing urease or GOx on the particle’s surfaces [71]. Moreover, Schattling et al. produced Janus particles employing the GOx/Cat enzyme pair on one side of poly(dopamine)-coated silica (SiO_2_/PDA) particles (800 nm). Hereby, the propulsion mechanism included the H_2_O_2_ production by GOx, which, in turn, was decomposed into oxygen by Cat [76].

#### 2.1.3. Bubble Propulsion

##### H_2_O_2_ as Fuel

The bubble propelled movement of nano- and micromotors relies on the recoil of produced bubbles, typically on only one side of the nano- and micromotors or inside of tubes or funnels. Hereby, the propulsion is induced by bubble formation, e.g., O_2_ during the decomposition of H_2_O_2_. One possibility to achieve a movement is the production of Janus-like particles. Addressing cancer therapy, Wu et al. designed polymer-Au Janus particles (7 µm) and attached catalase on the Au side. The catalase reacted with H_2_O_2_ inducing the recoil through O_2_ bubbles. DOX was embedded inside the particles during the polymerization process and could be released via near-infrared (NIR) irradiation. The directed, magnetic transport towards HeLa cells was achieved with the incorporation of Ni particles [79]. Xuan et al. covered one side of mesoporous SiO_2_ nanoparticles (75 nm) with Pt/Cr. DOX could be incorporated by physical adsorption and the addition of a folic acid component prevented a premature release of DOX molecules from the pores and supported the adhesion of the nanomotors on the surface of HeLa cells [80]. Wang and co-workers also presented a biohybrid approach, in which they half-coated a virus with Pt. The tamoxifen loaded nanoparticles moved in H_2_O_2_ and cell experiments were demonstrated on breast cancer cells [81].

A further approach of generating a movement is the use of nano-/microtubes or –funnels with different opening sizes. In 2009, the group of Schmidt introduced this concept of catalytic tubular engines [82]. The harvested fuels from the local environment enter the tube at the narrow opening and react inside the cavity, where the catalyst is located, to produce bubbles that extrude from the wider opening for the thrust. At the best, the outside of these engines consists of an inert material. By designing the composition, geometry and size of the rocket itself as well as the openings, the propulsion behavior of these engines is tunable. In the past, mechanistic prospects of the bubble release have also been investigated [83,84,85,86,87,88]. In that manner, Wu et al. utilized a layer-by-layer deposition technique to form nanotubes build up from chitosan (CHI) and sodium alginate (ALG), in which Pt nanoparticles were assembled. The resulting DOX-loaded (CHI/ALG)_18_-Pt nanotubes moved in H_2_O_2_ and iron oxide nanoparticles allowed magnetic guided attachment on HeLa cells. The detachment of the nanotubes was realized with ultrasound [89]. Furthermore, the same researchers utilized 30 µm rockets build up from bovine serum albumin/poly-l-lysine (PLL/BSA) multilayers in which catalase reacted with H_2_O_2_ to O_2_. Upon addition of magnetic particles, the DOX-loaded (PLL/BSA)10 -CAT-AuNPs-gelatin rockets could attach to HeLa cells and the drug was released via NIR irradiation induced gelatin melting. Moreover, these rockets were biodegradable by enzymatic, endogenous processes after delivering the drug [90]. Recently, Beladi-Mousavi et al. synthesized Bi/Ni/Pt microtubes (15 µm) which were propelled by O_2_ formed from H_2_O_2_ at the Pt layer inside the tubular microrobot. The robots were guided mechanically and could be loaded with DOX. The drug could be released electrochemically by applying a voltage, which did not affect the cancer cells as the experiments showed. Furthermore, the Bi/Ni/Pt microtubes were capable of readsorbing prior released, but unused DOX. Cell culturing experiments revealed the potential for improving the therapeutic effect when compared to free DOX [91].

Wilson and co-workers also designed bubble-propelled stomatocyte polymersomes, which moved through the thrust of O_2_ during the H_2_O_2_ decomposition at the surface of attached Pt inside the stomatocyte [92,93]. Additionally, DOX could be incorporated and guidance towards HeLa cells could be achieved magnetically [92]. The group also presented the biodegradability of these motors by altering the polymer composition [93]. Another interesting structure was exploited by Wang et al. The researchers loaded porous zeolitic imidazolate framework-67 (ZIF-67) with DOX and propelled these with O_2_ bubbles generated from H_2_O_2_. Their magnetic guidability was proofed and the drug could be released by adding H_2_O_2_ [94].

In general, a linear dependency of the particle speed on the H_2_O_2_ concentration was found [94,95] as well as a decrease of the number of active particles with decreasing concentration, e.g., in 30% H_2_O_2_ 76% of robots were active, in 0.2% H_2_O_2_ the number was reduced to ~25% [80]. According to the afore discussed possible toxicity of H_2_O_2_, also here alternative fuels were investigated.

##### Developments towards More Biocompatible Fuels

Water has been reported as biologically friendly fuel. Most importantly, Mg was presented as metal catalysts. Mg is an essential trace element and its dietary intake provides health benefits [96]. Mou et al. developed Mg/Pt [97] and Mg/Pt-PNIPAM [98] Janus micromotors with a size of 50 μm. Thereby, H_2_ is generated by the reaction of Mg with H_2_O in simulated body fluid (SBF). In both systems the removal of the Mg(OH)_2_ passivation layer on the Mg surface is achieved with endogenous chloride anions in SBF. The Mg/Pt-PNIPAM micromotors were capable of delivering drugs, which was shown with fluorescein (FITC) as model drug. The thermoresponsive PNIPAM shell enabled the uptake and release of the drug. Acid can be another fuel for driving nano- and micromotors. Typically, the tumor environment is acidic caused by the anaerobic glucose metabolism producing lactic acid [99]. Schmidt and co-workers utilized the extremely light acidic environment generated by HeLa cells to propel calcium carbonate/Co Janus particles (7 µm) [100]. Their speed was comparably low due to the low concentration. However, these micromotors are promising candidates for drug delivery in vivo since no external fuel is necessary.

Recently, Wan et al. presented a nanomotor driven by nitric oxide (NO) [101]. The hyperbranched polyamide/l-arginine (HLA) utilized l-arginine as fuel for the production of NO. The latter served as the driving force and as the anticancer component, which is yet to be demonstrated. The particles were self-degradable as could easily be imaged due to their fluorescence.

However, the in vivo utilization of bubble propelled nano- and micromotors is still under debate. In some biological environments bubbles are unfavorable. In this context, on the one side, an excess of H_2_ gas can lead to gas thrombus. On the other side, H_2_ can be used as therapeutic agent in cancer therapy. Therefore, H_2_ release could be beneficial [102]. Finding the compromise within this research field is an important objective. Ammonia, the degradation product of propulsion by urea, is as well considered being toxic to the human brain and cells [103].

Oxygen generally occurs in the body only when it is bound to hemoglobin. Free oxygen (e.g., in form of O_2_ bubbles) would lead to the generation of huge amounts of reactive oxygen species, which are toxic to the body. The solubility of oxygen is low and the formation of gas bubbles is toxic [104]. Bubbles exert pathophysiological effects by mechanically distorting tissues, obstructing blood flow and initiating an inflammatory response [105].

In general, the chemically-powered, often phoretic microswimmers show excellent performance and reproducibility. However, they present several drawbacks towards biomedical applications: The fuels that these micromotors rely on are often not biocompatible and their swimming behavior is often not easy to modulate, since on or off states depend frequently on the presence or absence of chemical fuel.

In order to be independent from chemical fuels and their related degradation products, externally propelled nano- and microengines have been studied. We will focus on these in the following section.

### 2.2. Energy Transferred by External Fields

**Figure 2:**
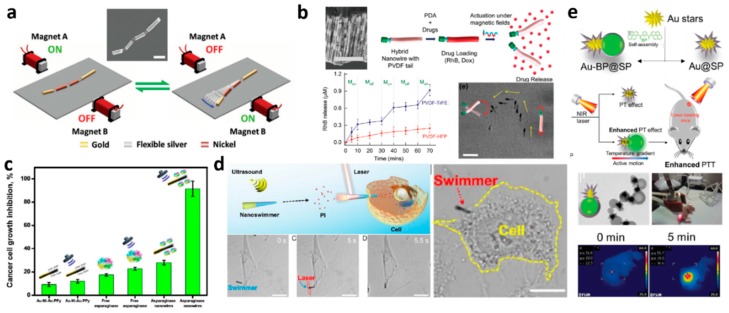
Summarizes selected examples of external field driven nano- and micromotors: (**a**) Magnetic propulsion of an artificial nanofish using a planar oscillating magnetic field, inset: SEM image of a multilinked artificial nanofish made by electrodeposition. Scale bar: 800 nm. Reprinted from [115] with permission from John Wiley and Sons, Copyright 2016. (**b**) SEM image showing alternating Ni–Au nano-ring segments grown around PPy NWs, Scale bar 4 µm, Time-lapse showing a single hybrid nanoeel transition from a surface-walking swimming mode to a wobbling motion upon changing the parameters of magnetic fields. Scale bars: 15 μm. Controlled drug delivery with the hybrid nanoeels starting from functionalization with PDA and drugs, followed by magnetically triggered drug release and pulsatile release of RhB with and without magnetic fields (*n* = 6), Reprinted from [43] with permission from John Wiley and Sons, Copyright 2019. (**c**) Cancer growth inhibitory effects of free and immobilized asparaginase compared to different control experiments, error bars show the standard deviation of 3 measurements (*n* = 3). Reprinted with permission from [137] Copyright RSC 2017. (**d**) NIR light-assisted cell poration. Schematic cell poration of the AuNS-functionalized (PSS/PAH) nanoswimmers upon the exposure of NIR light. Time lapse showing the movement of the nanoswimmers toward HeLa cell under the acoustic field and the perforation with NIR irradiation. Scale bars, 10 μm. Blue dash line shows the trajectory of acoustic driving and red circle indicates the region of laser spot. CLSM image of the nanoswimmers after cell poration. Yellow dash line indicates the frontier of the cell. Scale bar, 10 μm. Adapted with permission from [111]. Copyright (2019) American Chemical Society. (**e**) Schematic illustration of a self-propulsion Au-BP@SP Janus nanoparticle for cancer cell treatment under NIR laser irradiation. TEM images characterizing AuBP@ SP Janus nanohybrid. The Infrared thermal images of Au@SP and Au-BP7@SP injected MCF-7 tumor-bearing mice at different time points under laser irradiation at 808 nm. Reprinted from [142] with permission from John Wiley and Sons, Copyright 2016.

In this part, most presented nano- and micromotors are moving through interactions with different external fields. These motile systems have been denominated “constricted” or “driven” since they are not completely force and torque free and the definition “microswimmer” is not always agreed on. However, since most of these systems do not require the presence of chemicals for propulsion, their use is often seen very promising for medical uses. An overview of micromotors targeting cancer and driven by external fields are listed in Table 2 and representative examples are shown in Figure 2.

#### 2.2.1. Electric Fields

Electric fields by themselves are able to cause charged particles to undergo electrophoresis, which would not fulfill the criteria of self-propulsion. In combination with an applied frequency, resulting in an external AC electric field, certain material combinations give rise to Induced-Charge Electrophoresis (ICEP) and self-dielectrophoresis (sDEP). Both mechanisms are related and require metallodielectric Janus particles, but differentiate in the frequency of the electric field. At low frequencies, the different polarizability of two hemispheres after building up the electric double layer leads to an osmotic flow [106,107,108]. When the frequencies are increased beyond the relaxation time of the electric double layer, the Janus particles are driven by a dielectrophoretic force arising from localized electric field gradients generated between Janus particle and substrate moving in the opposite direction (sDEP). However, besides being very sensitive to material changes, and salt concentrations, these experiments require a complex electrode setup, which is unlikely to be ever used in real medical environments, for which there have also not been any proof of concept approaches.

#### 2.2.2. Magnetic Fields

Magnetic fields have been used for a long time to induce motility into systems. The use of magnetic gradients and the following magnetic attraction of magnetic objects is not considered a propulsion mechanism for a microswimmer. This scheme was presented for drug delivery using a hydrogel bilayer structure with encapsulated iron oxide particles (Fe_3_O_4_). One hydrogel layer consists of a pH-responsive gel, which traps or unfolds the soft microrobot depending on the pH, while the Fe_3_O_4_-encapsulating layer is responsible for the locomotion in the magnetic field [109]. This micromotor exhibits a preferred magnetic orientation. A uniform magnetic field gradient in the region of interest is required to propel the microrobot along the aligned direction and once reached the goal, the soft microbot opened and released the drug filled capsules. A similar approach on a larger scale was presented by the Sitti group [110]. The use of rotating fields in combination with mostly helical shapes is a sophisticated approach allowing for propulsion with high precision even in complex, often highly viscous, biological fluids. Xin et al. analyzed the shape optimization for both, swimming and transport capabilities [111]. Also liquid metals have been used as guidable objects [112] and motility control was implemented using a magnetic trap [113]. Generally, propulsion by magnetic fields can be divided into rotating and oscillating magnetic fields, with few examples of other strategies [114].

J.Wang’s team was inspired by the body and fin caudal motion used by fish and mimicked it using a magnetically-powered multilink nanofish consisting of different flexibly linked segments made of Au and Ni [115]. An oscillating magnetic field achieved a back-bending wave that propelled the microswimmer, which is envisioned for biomedical applications.

The motors based on rotating magnetic fields are more efficient and further developed: The first approach was made by Ishiyama et al. [116] and a few years later downscaled to the microscale by a team around B. Nelson [117,118]. A further downscaling step that brings these systems closer to the biological paragon of the bacterial flagellum was achieved by Ghosh et al. [119]. In vivo swimming of individual microswimmers has been proven in several publications, but to increase the impact many micromotors will have to be employed at once, as shown in the work of Servant et al. where a swarm of bacteria-like microrobotic flagella achieved deep penetration into a mouse [120].

Drug delivery approaches include a liposome-based temperature dependent release [121] and more specifically a cargo transportation strategy based on a rotating magnetic Ni head (≈1.5 μm long), along with a flexible Ag segment (≈4 μm long) which is coupled to drug-loaded magnetic polymeric microspheres of various sizes made of poly(d,l-lactic-co–glycolic acid) [57]. Interesting is, that the particles’ magnetic moment is several orders of magnitude smaller than that of the Ni segment and therefore the particle rotates jointly with the micromotor. The additional viscous drag and the rotating flow field influence the propulsion and therefore particles of different sizes lead to different changes of the motor speeds after pick-up. The potential practical utility of these fuel-free nanomotors was demonstrated by experimentally delivering DOX-loaded PLGA microparticles to cancer cells in a microfluidic chip, showing that the drug reaches the cells significantly faster than by pure diffusion.

A team from ETH Zürich has developed on-board drug transporting nanowires [122]. Later, a so called nanoeel, that resembles an electric eel because the soft cylindrical bodies composed of a flexible ferroelectric polyvinylidene fluoride based polymer linked to a polypyrrole nanowire with Ni rings [43]. A rotating magnetic field sets the swimmer into motion and deforms the plastic tail, strain is induced, changing the electric polarization through a piezoelectric effect. Tuning the magnetic parameters allows using this deformation either for propulsion or for drug transportation by surface functionalization with polydopamine. Swimmers with piezoelectric tails even enabled Mushtaq et al. [43] to selectively switch drug release on and off, a feature that was again demonstrated using cancer cells. That delivery does not need to be restricted to pharmaceutical compounds was shown using stem cells transported by micromotile systems [123,124].

The second interesting application for these, mostly rigid, microswimmers are mechanical interactions with target tissues that could be used to perform microscale surgery. Schmidt et al. proposed an innovative strategy to perform minimally invasive surgery [21] by using a rotating magnetic field to induce rotational motion on a sharp rolled up microtube, formed from a trapezoidal geometry. The removal of material from the body tissue is believed to benefit from the surface friction between tissue and microtube tip, resulting in a hole of a diameter close to the microtool dimensions.

A device that is slightly larger than the typical nano- or micromotor but able to combine targeted drug delivery and minimally invasive surgery was presented by Pané’s group [125]. This implantable microrobot was fabricated by a sophisticated electrochemical method which resulted in a tubular structure made of Ni and Co and could therefore be magnetically controlled, which was shown in vivo in the vitreous humor of a rabbit eye. The combination of soft and magnetic micromotors was presented by Sitti’s group [126].

Not in all cases complex technical devices are required for fabrication, Yan et al. for example produced helical microswimmers by dipcoating Spirulina microalgae in a solution of magnetite nanoparticles. These were able to move within a rodent stomach and showed selective cytotoxicity to cancer cell lines. Additionally, the microswimmers were able to degrade and their inherent properties facilitated imaging [127]. Such magnetic microswimmers based on different biological templates have been increasingly popular, with one reason being their estimated high biocompatibility and the rather easy fabrication. Further plant based examples with biomedical relevance are the medibots by S. Kumar [128] or the spore based micromotors suggested for detection of toxins [129]. For some of these systems, even the intrinsic flexibility of the biological molds is conserved, a feature rarely achieved for fully artificial systems [130].

#### 2.2.3. Acoustically Driven Systems 

The term acoustically driven mainly refers to ultrasound, i.e., frequencies between 20 kHz up to several gigahertz. These waves are well known for medical imaging as well as nondestructive testing. Ultrasonic waves can be manipulated, confined and focused and have also been used in this context for driving micromotors. Early systems were asymmetric Au nanowire containing a concave-end segment. Their working principle makes use of acoustic fields and sound waves that are differentially scattered through curved materials. Nadal et al. associated the motion to steady streaming stress produced over the asymmetric surface of a particle, but the actual energy conversion is a complex process, influenced by different phenomena: an ambient fluid flow is produced by the non-linearity in the acoustic field [131]. Additionally, an acoustic radiation force surges from stresses on the particle by the field and causes the migration of the particles towards a levitation plane and acoustic nodes. The largest influence on asymmetric particles has an acoustic streaming, which induces disturbance fields in the vicinity of the particle and therefore causes the self-propulsion [132]. These relatively innocuous and easy to manipulate forces were used by Soto et al. on different shapes, namely hollow metal shells and the influence of different material parameters was evaluated and compared to theoretical predictions. After exploring these basic features and comparing intensely with theoretical predictions, Soto et al. also demonstrated the ability to perform complex tasks such as capture and transport of multiple magnetic cargoes, and internalization as well as propulsion inside living MCF-7 cancer cells. Later, the same group presented an approach using Au-Ni-Au nanorods with nanoporous segments with a polymer coating for loading DOX. The coating facilitated electrostatic interaction with the drug and the nanoporous Au could be selectively heated through a photothermal effect caused by NIR light, leading to triggered release. The effect on HeLa cells was demonstrated [133].

Furthermore, mechanical interaction with cells was achieved with polymer multilayer tubes that were propelled by ultrasound [134]. When the Au segments of the tubes were irradiated by NIR light in vicinity of a cell, the tube accomplishes the photomechanical poration of the cell membrane. Upon opening of the cell membrane, the surrounding molecules percolated into the cell which let the authors assign a potential for intracellular drug delivery, artificial insemination and subcellular surgery to their system. Another tool for mechanical interaction, precisely for probing the response of living cells to internal mechanical excitation [135], was presented by the Mallouk group, who found that Au rods attach to the external cell surface, followed by internalization in case of prolonged exposure. The Au rods moved significantly slower inside the cells, which could be due to the increased viscosity or a possible loss of acoustic power due to damping. The controlled motion of the rods caused vortices which influenced the suborganelles within the cell, or some motors were even trapped within vesicular structures of the cell [136]. An approach to deprive tumor cells of their nutrients presented Uygun et al. Lymphoblasts are not able to synthesize asparagines but rely on their uptake from the blood stream to provide this essential amino acid. By adding asparaginase to micromotors and therefore enzymatically degrading the asparagins in the blood stream deprives cancer cells of this important nutrient and inhibits the growth [137]. After grafting asparaginase on the electroplated Au/Ni/Au/PEDOT-PPy-COOH nanowires, Uygun et al. could observe increased activity of the enzyme, as well as a growth inhibition on the lymphoma cells [137].

#### 2.2.4. Light

Light as driving force is easily switchable (allowing an on–off use), wireless energy transmission is possible and the energy content can be readily changed using different wavelength. However, the use of light within the human body is strongly restricted by penetration depth, so that many of the micromotors propelled by UV or blue light are not a realistic approach for cancer therapy. Thus, we omit here most photocatalytic swimming strategies. Other biomedical applications have been discussed in a recent review [35].

The heat driven swimmers, first proposed by Würger theoretically [51], were then experimentally realized by Jiang [138]. Here, the absorption differences of the two hemispheres trigger the thermophoretic mobility in the thin interfacial layer and subsequently the particle motion [139]. However, similar to the other phoretic mechanisms, thermophoresis is strongly salt dependent and therefore difficult to implement in any biomedically relevant setting, due to the high ionic strength that prevail there. 

While most light-driven systems still rely on short wavelengths due to their high energy content, a few systems are already propelled by more biomedically relevant light sources like infrared (IR) or NIR irradiation. On the other side, promising approaches such as phototherapy, i.e., the heat transfer from irradiated Au nanoparticles to a surrounding medium by electron-photon relaxation and a following local temperature increase have also been combined with the principle of active motion. In a particle based approach the photothermal effect could also be triggered using a NIR laser and sufficiently small particles to achieve a long retention time in the body were used [140].

Wu et al. produced catalytic polymer-based multilayer microtubes, Pt nanoparticles inside and a thin Au shell on the outside, functionalized with tumor-targeting peptides as well as an antifouling PEG. In this publication the motion, despite being caused by the photothermal effect, still required a low concentration of peroxide. An increase of the light intensity caused an additional rise of the temperature and killing of cancer cells that were targeted by the peptide. The authors observed a photothermal motion in absence of peroxide, which makes them optimistic that with further tuning, this system could be independent of the peroxide fuel making the application of self-propelled synthetic engines in biomedical fields more realistic [141].

Yang et al. used Au nanostars coupled to organic bis-pyrene nanoaggregates coated with a PEG- functionalized silica shell. These composites showed thermophoretic motion, but also photothermal effects after NIR irradiation. The Janus nanohybrids had higher cell killer efficacy than Au@SP at the same concentration, probably because the active motion enhances the temperature of the treated cells even further, leading to better results [142]. Xuan et al. used the traditional Janus geometry made of mesoporous silica in combination with a Au shell, while the inert half is covered with macrophage cell membranes to increase speed and circulation time, overcome biofouling and enhance target recognition of on cancer cells. The Janus particles were able to thermomechanically percolate cell membranes under NIR light, which is proven by following the membrane rupture using propidium iodide [143].

Generally, using an external field as driving force has the advantage of being relatively insensitive to external conditions, avoiding many of the drawbacks found for chemical nano- and micromotors. On the other side it requires rather large and complex equipment to create the employed stimuli. One way around that is taking benefit of evolutionary wisdom, as discussed in the following section. 

### 2.3. Biological Constituents for Nano- and Micromotors

**Figure 3:**
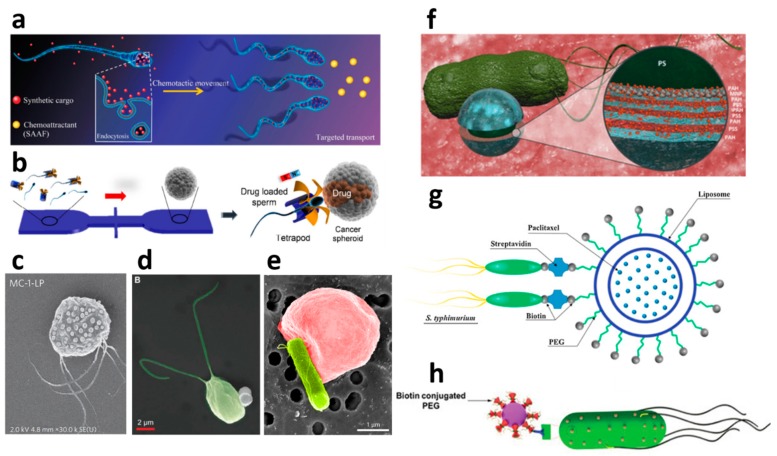
Biological propulsion units for anti-cancer therapy by nano-and micromotors: (**a**) Chemotactic sea squirt sperm loaded with cancer drugs and Pt nanoparticles (Chen, Adv. Biosyst. 2018) [144], Copyright 2018. (**b**) Bovine sperm loaded with DOX and captured in tetrapod-like structures (Xu et al. 2018) [145], Copyright 2017. (**c**) Magnetotactic bacteria decorated with drug-loaded nanoliposomes Reprinted by permission from Springer Nature (Felfoul 2016) [40] Copyright 2016. (**d**) Algae C. reinhardtti attached to magnetic polystyrene particle. Reprinted with permission from John Wiley and Sons, Yasa et al. Adv. Mat. 2018) [146] Copyright 2018. (**e**) Bacteria-propelled red blood cells which are loaded with DOX (Alapan Science robotics 2018) [147] Reprinted with permission from AAAS. (**f**) *E. coli* with polyelectrolyte multilayer microparticle loaded with DOX: Park et al. ACS Nano2017 [148], Reprinted with permission from Park et al. ACS Nano 2017 (**g**) Liposomal bacteria-based microrobot. Reprinted from Nguyen et al. [149] with permission from Elsevier. Copyright 2016. (**h**) *S.* typhimurium NanoBEADS, Suh et al. Adv. Sci. 2019 [150] Reprinted with permission from John Wiley and Sons, Suh et al. Adv. Sci. 2019, Copyright 2018.

Biological components have been very attractive approaches for biomedical applications of micromotors in recent years. There are various obvious reasons, such as biocompatibility, operation under physiological conditions, efficient motion on the microscale, interaction with living matter and other specific biological and pharmaceutical interactions. Here, we want to point out three important ways that biological entities have been employed for nano- and micromotor-based cancer therapy: as structural, loading and as propulsion units. Since we structured this review according to propulsion sources, all chemically or magnetically driven biohybrid motors can be found in Section 2.1 and Section 2.2. However, the features of the different biological loading units are briefly pointed out here. Up to date, blood cells, bacteria, viruses, plant tissue, macrophages and sperm cells have been incorporated as loading units for cargo delivery by micromotors. Representative examples of biohybrid micromotors targeting cancer are displayed in Figure 3 and an overview of the approaches is listed in Table 3.

#### 2.3.1. Structural Units

Using biological components as templates for the fabrication of nano- and micromotors is an attractive approach because it often provides high biocompatibility, degradability and simple fabrication. Since the structural and morphological variety of microobjects in nature is vast, researchers find inspiration and resort to natural templates for the fabrication of nano-and micromotors. The helical, rigid vessel of plants have been used as template for magnetically propelled micromotors by simple deposition of magnetic materials onto their surface [151]. Plant-based calcified microtubes were, in another approach, coated with iron and loaded with the anti-cancer drug campothecin [128]. Rotating magnetic fields allowed the drilling of the microtubes into cancer cells and thereby delivered the drug directly into the cells. Similar to plant structures, microalgae were used as templates for magnetic microswimmers. They were dip-coated in fluids containing magnetite nanoparticles, which results in biohybrid magnetic microswimmers. This approach allowed in vivo imaging of the micromotor swarms by magnetic resonance imaging [127].

Further, the magnetization of the algae *C. reinhardtii* was implemented by internal accumulation of terbium and thereby creates a magnetotactic micromotor [152] Non-motile bovine spermatozoa can also serve as template for the simple fabrication of flexible magnetic micromotors. Due to the electrostatic self-assembly of positively charged iron oxide particles and negatively charged sperm cells, a wide coverage of particles onto the cells is achieved [130]. In another study, the drug-loading ability of sperm cells was demonstrated [145], so that this biohybrid system holds promise for anti-cancer therapy.

#### 2.3.2. Loading Units

Cytotherapy, referring to the use of cells as payload carriers, is a novel approach in cancer therapy [153]. This idea is also being explored in the application of biohybrid nano-and micromotors, as discussed in this section.

##### Blood Cells

Red blood cells are referred to as “supercarriers” [154,155,156,157], because they display extraordinary properties as drug loading units. Drug loading has been demonstrated into their interior or onto their surface. Red blood cells are predestined to swim in the blood stream, so it is obvious that they display great promise for targeted cancer therapy. These cells can immensely improve the pharmacokinetics by prolonged circulation times of the loaded drug and are especially interesting for therapeutic targets that are accessible to red blood cells in the intra- and extravascular system. The drug clearance is hence not dependent on the renal filtration, but rather limited by hemoglobin degradation.

Red blood cells have been incorporated into biohybrid micromotors, by loading them with DOXand magnetic nanoparticles which allows the magnetic guidance and propulsion by ultrasound, as well as reduced toxicity of the drug when encapsulated in the blood cells [141,158]. In addition, photosensitizers were loaded into red blood cell-mimicking micromotors for demonstration of photodynamic therapy by the generation of oxygen radicals or photothermal chemotherapy in combination with NIR irradiation [159,160,161,162].

Other blood cells such as neutrophils or lymphocytes (T cells) have been shown to work for cytotherapy as well, by loading them with anti-cancer drugs [163] or Au nanoparticles and applying photothermal therapy [164], respectively, but the propulsion is strictly speaking passive.

Macrophages are phagocytotic white blood cells that are responsible for the immune response by engulfing cancer cells and can therefore be considered an anti-cancer strategy that relies on the digestion of single cancer cells. Macrophages have been combined with Mg Janus particles to create biohybrid motors that are propelled in acidic medium while maintaining full cell functionality [165]. Thus far, biohybrid macrophage-micromotors have been demonstrated to neutralize bacterial endotoxins [165]. It is conceivable that macrophages will play a role in cancer-targeting micromotors in the near future due to their unique ability to digest cells while doing no harm to healthy cells and being the human body’s natural defense. Furthermore, tumor-homing (the targeted transport to the location of the tumor) has been applied by the use of magnetic nanoparticle-loaded monocyte/macrophage-like cells [166]. The particle-loaded cells were injected intraperitoneally and migrated to the tumor. Local hyperthermia-treatment allowed then targeted tumor treatment while increasing survival rate in the murine pancreatic cancer model. Even though this approach does not include actively moving components, it delivers nanoparticles in a very targeted way.

##### Viruses

Virus-based drug delivery is a new approach to tackle cancer and has so far only once been utilized as micromotor components by Wang’s group [81]. Hereby, plant viruses have been loaded with the cancer drug tamoxifen and covered by a Pt layer to create a Janus like self-propelled virus. Viruses have previously been used as bioactive coatings of drug delivery systems [167] employing their various benefits. Due to their small size (tens of nanometers), viruses are very attractive, because they are small enough to move through the bloodstream, nontoxic and able to enter cells [168]. Viruses are also good candidates to protect poorly soluble drugs while offering good biocompatibility during their transport. Genetic and chemical modification enables the addition of specific functionalities and tissue-specificity which allows the effective uptake by cancer cells. For instance, cancer cells show upregulation of certain receptors on their surface. When viruses are genetically modified to show receptor-binding units on their surface, a specific uptake by cancer cells and avoidance by healthy cells has been achieved [169,170,171]. Plant viruses can be produced by low costs in monodisperse solutions and are therefore attractive alternatives to synthetic loading units. 

##### Spores 

Spores have not been utilized for drug delivery so far, but show potential for toxin-sensing by functionalization with iron oxide particles and carbon dots [129]. Plant spores such as the spiky sunflower pollen capsules have also benefits due to their hollow structure for uptake of fluorescent BSA as model agent and their rough and spiky outer surface with is an excellent template for Pt coating and bubble propulsion in peroxide solutions [172]. However, the prospects of these micromotors based on spores are limited in anti-cancer therapy due to their toxic propulsion mechanism.

#### 2.3.3. Propulsion Units

##### Bacteria

Motile bacteria are useful units for the propulsion of biohybrid micromotors. Their single or multiple motile appendages allow the transport of the attached cargo. The multifold taxis abilities of bacteria offer many guidance and control mechanisms. Up to now, chemotaxis [149,173,174,175], aerotaxis [40], magnetotaxis [40,55,176] and pH-taxis [177] have been explored, leaving much freedom for future exploration of control by thermotaxis, phototaxis, thigmotaxis, galvanotaxis, just to name a few. Another nice feature of using bacteria is the ability to genetically engineer their properties.

In initial works on bacterial biohybrid micromotors, particles were attached to motile bacteria by adsorption [53,178]. Bacteriabots were also fabricated by attaching micro-or nanoparticles via biotin-streptavidin conjugation [179,180]. Later on, anti-cancer therapy was proposed with magnetotactic bacteria that were decorated with drug-containing nanoliposomes [40]. These motile bacteria were directed to the hypoxic regions of the tumor sites by aerotaxis. In this case, the bacteria function as magnetic constituents, propulsion units and loading units. Nguyen et al. attached *Salmonella* typhimurium to liposomes via streptavidin-biotin affinity and thereby demonstrated cancer-therapeutic bacteriabot [149]. The liposomes were loaded with paclitaxel as a tumor-targeting agent and their cytotoxicity on breast cancer cells was compared to drug-loaded liposomes without bacteria. Although no directed guidance mechanism was used, the longer and faster movement of the drug-carrying bacteriabots caused a significantly higher therapeutic effect than non-motile drug-loaded liposomes. The bacterial actuation led to enhanced diffusion of the released drug. 

Further, motile *E. coli* bacteria were attached to the surface of drug-loaded polyelectrolyte multilayer microparticles [148]. Embedded magnetic nanoparticles allowed the magnetic control of the bacteriabots, while the loaded DOX targeted breast cancer cells in vitro.

An interesting approach was made by combining red blood cells with bioengineered *E. coli* as propulsion units [147]. The red blood cells were filled with superparamagnetic iron oxide nanoparticles for magnetic guidance and DOX for targeted drug delivery. Its biological propulsion and loading units are beneficial due to their biocompatibility, degradability and ability to squeeze through tight spaces. A similar approach was made my combining microemulsions with *E. coli* [181]. This biocompatible and bacteria-based micromotor is also able to squeeze through tight spaces and avoids any damage to the surrounding tissue owing to its softness. 

Another specific advantage of using bacteriabots was found due to their adhesion ability to epithelial cells by bacteria type I pili and mannose molecules on the epithelial cells [182]. *E. coli* strains grow type I pili on their surface, which contain mannose-binding units. Pathogenic *E. coli* use this mechanism to infest the urinary or intestinal tract. This bioadhesive drug carrier enhances the targeting of the drug-loaded particles which are carried to disease site by the bacteria. However, the bioadhesive bacteriobot cannot distinguish between healthy and cancerous tissue.

Apart from attaching drug-loaded particles to motile bacteria, bacteria can also be captured inside electropolymerized microtubes for magnetic directional control [183].

Even if bacteria have up to now only been used as propulsion units, their ability to fight cancer is a rising topic [184]. Bacteria can be engineered to have several anti-tumor activities, such as apoptosis induction, autophagy, bursting of invaded tumor cells or by secretion of exotoxins [185]. As an example, motile *E. coli* bacteria were directly linked to DOX by acid-labile linkers and showed increased accumulation of DOX in the tumor, thereby enhancing the temporal and spatial control of the drug action [186]. Bacteria such as *S.* typhimurium are known to accumulate and replicate in solid tumors [184]. Trials towards the application of these bacterial anti-tumor features were demonstrated by Suh et al. with bacteria-enabled autonomous nanomotors. *S.* typhimurium conjugated to nanoparticles were shown to penetrate regions far from the circulation without any external driving force [150]. The self-replication and translocation due to the bacterial motion effectively delivered nanoscale loads. These characteristics might become interesting in future applications of bacteriabots.

##### Sperm Cells

The male germ cell has just recently been employed as both, propulsion and loading unit for anti-cancer therapy by micromotors. The first demonstration was done by Xu et al. [145] using bovine sperm cells and a tetrapod-like artificial structure that allowed the magnetic guidance and mechanical release of the sperm cell at the location of the cancer spheroid. The drug-loaded sperm cell then attached to the cancer cells and released the drug. This was also the first time that spermatozoa were demonstrated to function as drug loading units, while their motility remains largely unaffected. Lately, the drug loading of human spermatozoa was also implemented, making the spermbot-mediated drug delivery more suitable to be transferred to clinical therapy for gynecological cancer [187].

Spermatozoa from the sea squirt Ciona were loaded with various agents, DOX amongst others, and guided to ovarian cancer cells [144]. The sea squirt is an external fertilizer, thus, chemotaxis can be used for directed motion in a gradient of sperm activating and attracting factors that are released from the eggs. The additional agent such as fluorescently labelled Pt nanoparticles and quantum dots allow additional potential applications such as hyperthermia, biomolecule detection ad in vivo imaging. The limiting factors of the drug loaded sea squirt sperm cells is that they are adapted to swim in sea water and therefore, it will be difficult to adapt the concept of chemotactic guidance to human or mammalian sperm, in which chemotaxis as guidance mechanism might only work over very short distances in the close distance from the egg.

##### Algae

The freshwater green microalga Chlamydomonas reinhardtii was demonstrated to serve as propulsion unit for magnetic polystyrene particles [146]. As a model drug, fluorescently labelled isothiocyanate-dextran was loaded onto the polystyrene particles and delivered to mammalian cells. Even though this example is not targeting cancer, it is supposable that this biohybrid system could work in anticancer therapies. The propulsion of the algae-driven micromotors were tested in several relevant fluids such as human tubal fluid and different cell culture media.

### 2.4. Shape-Changing Nano-and Micromotors for Cancer Therapy 

One rather new part is the idea of emulating shape changing biological swimmers. The deformation of bodies for forward motion is being using by many organisms, e.g., slime molds or jellyfish [188,189]. In general, soft and deformable micromotors display prospects in many fields, as discussed recently [190]. This principle has been realized on the macroscale [189], and was introduced on the microscale by the Fischer group by mimicking the motion of a paramecium [24]. A photoactive, liquid-crystal elastomer, worm-like micromotor is actuated by exposure to structured light which leads to travelling-wave motions to self-propel. Furthermore, thermoresponsive, polymeric strips are propelled by laser pulses that cause a repeated reconfiguration of the strips [25]. These examples display propulsion based on shape change, but no drug delivery application was demonstrated so far. In contrast, microgrippers that pick and place based on shape changes, but need magnetic force to be moved, have been shown to function also as therapeutic agents [191,192]. Malachowski et al. fabricated a thermoresponsive microgripper that can be loaded with DOX and released the drug in the porcine stomach [193]. In a similar manner, microgrippers showed to release alginate particles on demand by NIR-triggers [194]. Kobayashi et al. demonstrated magnetically actuated, thermoresponsive and biodegradable grippers with ability for drug delivery [195]. Moving to slightly larger scales, these systems can handle more advanced functionalities [110]. and there have been many publications on several different approaches over the last decade [196,197].

Overall, up to now, none of the micromotors propelled by shape change have been used for active anti-cancer therapy. However, the ability to change the shape allows for instance the movement through tight spaces or bottlenecks where other, rigid micromotors might fail because they get stuck. Emulating shape change like living organisms is a fascinating route for future approaches to develop smart and adaptive micromotors.

## 3. Performance Optimization

### 3.1. Power Transmission

Since on board fuel storage is limited due to the small dimensions of nano- and micromotors these need to harvest energy from their surroundings. As discussed in the previous section, many different approaches have been proposed to this end. But once motility is achieved, the work is far from done, there are rather several further steps to be mastered:

### 3.2. Loading

The options to deliver therapeutically relevant components are not limited to drugs. Also cells, microtools or genetic materials might need to be transported to specific sites within the body. However, depending on the type of cargo, different loading strategies can be applied. One of the easiest methods is simply attach the component via physical adsorption [80] which on the other hand does mostly not allow very controlled dosing or further handling. Here, surface coverings might increase the interactions by facilitating also electrostatic attractions. An often employed attachment are magnetic interactions, which have been used frequently for micromotors but require specific properties of both, carrier as well as cargo [57].

Even though encapsulation strategies have been used in the field of micromotors [89], much more sophisticated methods have been developed separately [41,42,198] and will most likely soon be implemented into novel micromotor structures. The question how loading mechanisms can be improved in order to become not only stable in different environments but at the same time allow complete on demand release independent of the propulsion mechanism still requires investigation.

### 3.3. Guidance

In principle, all guidance mechanisms that have been used in laboratory settings are applicable to biomedical applications as well. One main guidance mechanism to be pointed out is magnetism, that is frequently used for most types of micromotors, ranging from bacteria, to Janus particles, bimetallic rods and bubble driven microtubes. However, also other types of guidance mechanisms, (e.g., surface interactions [199]) could potentially be used in specific settings.

Especially promising in biological environments is the use of tactic behaviours that are frequently found in microorganisms [200]. Biologically relevant environments offer various gradients that can be explored, far beyond the movement towards a fuel or a pH gradient. Directly related to this, a H_2_O_2_ gradient is frequently produced in tumor tissues [65], which has already been explored by nanorobots following these [61,201].

In particular, the hypoxia region in a solid tumor has distinctive properties and creates gradients in its microenvironment. One property is the very low oxygen concentration and a deficiency in nutrients [40,202] caused by the increased metabolism of the cancer cells. Similar effects have been observed concerning the local pH value [177] and used for the direction of carbonate based microswimmers [100].

### 3.4. Release

In most cases, the cargo release is triggered passively when the nano- or micromotor swims through local changes in physiological conditions like pH or temperature, that are often associated to tumors. The physiological conditions, however, are very complicated and often lead to unexpected changes that could trigger an uncontrolled release of the drug. Therefore, externally triggered release strategies have been presented, which often make the design of the nano- and micromotors more complex. Importantly, the drug releasing stimulus should be uncoupled from the propulsion source to avoid unwanted interference. 

NIR light is attractive for biomedical applications because body tissue has the highest transmissivity in this region (=biological window), but is still limited to a few millimeters. In-situ degradation of the drug-loaded material is one option, e.g., demonstrated by Tu et al. on stomatocytes with disulfide bonds of block-copolymer. Upon reaction with the endogenous reducing agent glutathione, the disulfide bonds break and DOX was released [61]. Another chemical approach of triggered drug release is the encapsulation of a drug-loaded nano- and micromotor and the selective capsule dissolution. For example, DOX-loaded mesopourous SiO_2_/Cr/Pt nanomotors were coated with an egg phosphatidylcholine (PC) bilayer and intracellular phospholipases triggered the catalytic hydrolysis of the shell [80]. Furthermore, Wu et al. utilized the photothermal effect of Au nanoparticles on catalytically propelled bovine serum albumin/poly-l-lysine (PLL/BSA) microrockets. These micromotors were fabricated with layer-by-layer deposition into a template. The final micromotors contain a thermoresponsive layer of gelatin and Au nanoparticles. When the photothermal effect induced by NIR laser irradiation leads to the heat-up and melting of gelatin, the drug is released [90]. The use of a photocleavable linker molecule between the micromotor and the drug also provides the possibility to release the drug upon light-irradiation [203].

Furthermore, alternating magnetic fields were utilized for targeted drug release: either via coupling between the piezoelectric and magnetostrictive phases [122] or relying on pick-up and release of drugs loaded on to cargoes such as polymer particles or liposomes [57].

The electrically triggered drug release has been proposed as well. One possibility is the electroreductive release by applying a constant potential in order to create a negative repulsion between the cargo (DOX) and the micromotor [91]. Further, nanomotors can be mechanically rotated via a rotating electric field, which enables the release of biochemical molecules in a controlled manner. The rotation speed of such structures is correlated with the amount of released cargo [204].

The use of ultrasound to externally direct a drug release as has been demonstrated on Pt/CHI/ALG)_18_ nanocarriers, which collapsed after ultrasonic treatment and released the DOX [89].

In the context of sperm-driven micromotors, the drug release was triggered via a sperm-cancer cell-fusion leading to apoptosis of cancer cells after the drug has been release [145]. Generally, cell-based drug carriers offer biological release mechanisms based on by cell-cell-fusion, endocytosis, phagocytosis or other entry pathways. The mechanisms employed in cell-based micromotor applications can be improved further according to knowledge from intracellular drug delivery studies, that point out that size, shape and surface properties, amongst others, play a crucial role for the efficiency of the drug delivery [205].

### 3.5. In Vivo Imaging

Generally, videomicroscopy is used to characterize micromotors but due to the non-transparency of body tissue to visible or UV light, this method is limited in vitro experiments. Therefore, nano- and micromotors require high resolution (micrometer) imaging techniques that work in real time and with deep penetration of targeting biomedical applications such as cancer therapy [206,207,208]. Since these reviews give very detailed information on all recent developments Fluorescence-based imaging in combination with dyes like FITC or directly using fluorescent drugs like DOX are frequently used in micromotor research [79,98], we only introduce the topic. For in vivo imaging the penetration depth of the radiation into tissue should be taken into account, for which the NIR spectral region seems to be one of the most suitable approaches [209,210]. Even though the fluorescence efficiency is constantly improved, the resolution on the microscale still needs to be improved to be applied to micromotors. Servant et al. managed however, to control and precisely locate a swarm of magnetically driven Ni/Ti helices, which were anodized and subsequently functionalized with a NIR 797 dye [120].

Non-optical methods, are frequently used in medicine with one example being MRI. Some of these, have been tried on micromotors and were found to be useful, others did not yet have enough resolution. MRI can for example be used in particular combined with paramagnetic and ferromagnetic particles to give MRI signal contrast. However, for magnetically driven swimmers, MRI becomes unfeasible because the high magnetic fields interfere with the propulsion.

Another medical technology that was applied for micromotors is positron emission tomography (PET), a molecular imaging technique that requires uptake of positron emitting isotopes. This principle has been shown by Vilela et al. on a large population of tubular Au/PEDOT/Pt micromotors, bearing an iodine isotope onto the micromotor’s Au surface. 

Iacovacci et al. used nuclear medical imaging (single-photon emission computed tomography (SPECT imaging) to image single soft microrobots with 100 µm dimensions inside the mouse [211]. 

Multispectral optoacoustic tomography is another approach for deep tissue imaging of micromotors that allows real time imaging of single micro-objects below centimeter thick tissue-mimicking phantoms [212].

Ultrasound is a technique that is well established in the medical field and considered a safe, non-invasive method that allows deep tissue imaging with moderate resolution. Preliminary trials of imaging micromotors with ultrasound has been demonstrated by tracking the bubble tails of catalytic microjets or 100 µm sized paramagnetic particles [213,214]. Thus, this technique is promising for swarms or even single object tracking, but needs further refinement regarding echogenicity of the micromotors, signal amplification and image processing. Photoacoustic effects have been used for both, in-vivo imaging as well as navigation [215].

### 3.6. Retrieval of Micromotors

A great challenge ahead will also be the development of an efficient way to remove micromotors from the body after their task is completed. This aspect is important in order to reduce the side effects and long term effects of the nano-and micromotor-mediated drug therapy. One simple approach could be to remove the micromotors in the same way that they were introduced to the target site, using their self-propulsion. Another approach that is discussed often, but seems rather impractical for blood, is filtering out the nanomotors from the blood stream. Body fluids contain several colloidal compounds, so that this approach seems however, more suitable for environmental remediation, where less solid compounds in the microscale are in the swimming medium. Realistically, the cost and invasiveness of filtering actions mostly inhibit their use.

The probably most promising retrieval strategy that has been presented so far is based on magnetic accumulation. Iacovacci et al. [216] demonstrated an intravascular catheter that is able to retrieve magnetic nanoparticles from the bloodstream. The device consists of a miniature module, based on 27 permanent magnets arranged in two coaxial series, integrated into a catheter. With this device, the majority of the unused circulating agents (500 and 250 nm superparamagnetic iron oxide nanoparticles) was shown to be removed from the bloodstream. 

Another approach for micromotor retrieval was demonstrated by Yim et al. by a magnetic capsule endoscope with wet adhesive patch [217]. This capsule contains a delivery unit for the release of microgrippers. On one end of the biopsy capsule, a retrieval unit is installed which contains a hydrophobic surface due to microposts and viscous silicone oil. It collects the microgrippers based on hydrophobic interactions and traps the microgrippers due to viscous forces. 

A durable alternative to retrieving nano-and micromotors is the fabrication of fully biocompatible micromotors. This is a challenge, especially because many prototypes contain magnetic material that cannot be degraded. However, small amounts of inorganic material inside the human body are probably not harmful and can be taken up. The degradation or resorption of materials would also be the least invasive method, ideally, with non-toxic products while maintaining full functionality [126,218].

A few examples have gone into this direction, especially in the group of soft micromotors, e.g., protein-based microrockets [90] or polymeric microtubes [219], although degradation studies under physiological conditions are often still lacking. A recent example of a Marangoni swimmer made from squid-derived proteins was demonstrated to fully degrade in slightly acidic environment [220]. Further, enzymatic degradation can be achieved [90]. It remains the question what the long term effects of the degradation products on the body are, especially any inorganic matter that is not decomposed.

## 4. Outlook

Overall, recent advances of nano-and micromotor design and function indicate several key points to enhancing targeted cancer therapy, as illustrated in the previous paragraphs of this review. 

Besides all steps that have to be optimized for the process of efficient drug delivery in vicinity of the cancer cells, there are also some systemic issues that need to be taken into account when thinking beyond the proof of concept.

First of all, biocompatibility and non-toxicity of all compounds have to be guaranteed. By now most of the nano- and micromotors are just optimized for functionality, but not in terms of innocuousness. Except for micromotors that are intended to have an additional function in surgery not cutting, soft materials will need to be more frequently introduced to avoid unintended tissue damage. 

In general, the average micromotor size is about an order of magnitude larger than the average passive drug delivery vehicle. This might be advantageous for the use of complex fabrication techniques and for outswimming Brownian motion, but tissue penetration as well as taking benefit of the EPR (enhanced permeability and retention effect, i.e., small particles tend to accumulate preferentially in tumor compared to normal tissues) [221] is strongly limited to the lower nanoscale. Here, the community should join forces with researchers from the community of targeted drug delivery to implement features like valves for controlled release, advanced encapsulation techniques or release triggers. Up to now rarely considered in the micromotors community is the immune response or immunogenicity that intruding entities generally cause when entering the body. Zhang et al. have presented an approach using external macrophage layers [165], but more systematic studies are clearly needed. One system that is more tolerant in that regard is the gastrointestinal tract in the body. A few in vivo studies employing micromotors have been implemented in that environment [31]. As for now, drug delivery towards cancer cells has only been proven in vitro and difficulties may arise when expanding the experiments to in vivo applications. However, gastric delivery could be a promising route for delivering drugs to tumors in the digestive tract [37].

Currently, the study of collective interactions among micromotors [222,223,224] and their environment [225] is of rapid development in experimental and theoretical soft matter and has the potency to multiply the efficiency of an individual micromotor. Few studies already took that into account [120], but the controllability of the individual versus the swarm will impose many challenges. Attempts to control multiple micromotors individually have been recently presented on a larger scale [226].

One of the main issues when going towards biomedical applications is however, not only to show elegant proof of concept works, but upscalable, reproducible and reliable systems that can be mass fabricated with consistent properties and performances. These systems require a shelf life long enough to be available and safe in clinical scenarios, easy to apply and inexpensive. Once these challenges are overcome, the community of micromotors can turn towards further challenges, which are currently impairing therapeutic success. Here, mainly the formation of metastasis should be mentioned. One could imagine micromotors hunting for circulating cells that spread out in the body, but to the best of our knowledge this has not been adressed. Another problem in cancer medicine is the involvement of the immune system, which is very often compromised by the aggressive therapy. First materials that inhibit attacks of the immune system have been used, but much more in depth studies are required here. Alternatively, micromotors could also support the immune system in fighting the cancer but these strategies have yet to be developed. A different but related topic that is very promising is diagnostic sensing, both, in vivo and in vitro. However, in order to stay within the scope of this review, we refer the interested reader to some recent summary work by other authors [16,227,228]. Realistically seen, one needs to conclude that, not only for cancer treatment, but in order to enter any clinical application of micromotors, we still have a long way to go. On the other side the promises and hopes resting on active drug delivery and targeting cancer cells are many and the potential gain justifies intense research programs and the ongoing dedication of many PhD students.

## Figures and Tables

**Table 1:** List of phoretic and bubble-propelled nano-and micromotors for cancer therapy.

Carrier Material	Drug	Target	Propulsion	Guidance	Release	Speed [µm/s]	Ref.
*phoretic propulsion*							
Ni/(Au_50_/Ag_50_)/Ni/Pt nanowire	DOX on PLGA particles and liposomes	-	self-electrophoresis (5% H_2_O_2_)	magnetic	drag force	9	[57]
polymersome stomatocyte/Pt	DOX	HeLa cells	self-electrophoresis (4.98 mM H_2_O_2_)	chemotactic, H_2_O_2_ gradient	glutathione assisted	35	[61]
PM/Pt microparticles	DOX	T47D cells	diffusiophoresis (2.5wt% H_2_O_2_)	magnetic	diffusion	1.25	[62]
mesoporous SiO_2_/urease	DOX	HeLa cells	diffusiophoresis (urea)	-	urea supported	-	[70]
SiO_2_/PEG/urease	anti-FGFR3	bladder cancer cells	diffusiophoresis (urea)	*via* anti-FGFR3			[74]
*bubble propulsion*							
(PSS-PAH)_5_/Ni/Au/CAT	DOX	-	O_2_ (0.5% H_2_O_2_)	magnetic	heat (NIR light)	25	[79]
mesoporous SiO2/Cr/Pt	DOX	HeLa cells	O_2_ (0.2% H_2_O_2_)	-	catalytic hydrolysis of lipid bilayers	3.6	[80]
Virus/Pt	tamoxifen	MDA-231	O_2_ (1.5% H_2_O_2_)	acid-supported	-	4.15	[81]
Pt NP/(CHI/ALG)_18_ nanotube	DOX	HeLa cells	O_2_ (H_2_O_2_)	magnetic	ultrasound	22	[89]
(PLL/BSA)10 -CAT-AuNPs	DOX	HeLa cells	O_2_ (0.5% H_2_O_2_)	magnetic	NIR light, gelatin melting	4	[90]
polymersome stomatocyte/Pt/Ni	DOX	HeLa cells	O_2_ (1.5% v/v H_2_O_2_)	magnetic	-	12.4	[92]
polymersome stomatocyte/Pt	DOX	HeLa cells	O_2_ (4.98 mM H_2_O_2_)		acid buffer	39	[93]
ZIF-67	DOX	-	O_2_ (1% H_2_O_2_)	magnetic	through H_2_O_2_	15.32	[94]
CaCO_3_/Co	-	HeLa cells	CO_2_ (acid)	-	-	0.544	[100]
Polyamide/L-arginine (HLA)	particle itself	MCF-7 cells, HUVECs	NO (L-arginine and 20% H_2_O_2_)			3	[101]

**Table 2:** List of field driven nano- and micromotors for drug delivery.

Carrier Material	Drug	Loading Mechanism	Target	Propulsion	Guidance	Release	Speed [µm/s]	Ref.
FeGa@P(VDF-TrFE) nanowires	Paclitaxel	polydopamine functionalization	MDA MB 231	magnetically	piezoelectric	alternating magnetic field	-	[122]
Hydrogel grippers and polycaprolactone particles	docetaxel (DTX)	Closing the gripper	/	magnetically	magnetic	Opening the gripper	-	[109]
Au-Ni-Au nanorods with nanoporous segments	DOX	Electrostatic interaction with the drug	HeLa cells	ultrasound	magnetic	Photothermal (NIR)	60	[133]

**Table 3:** List of biohybrid nano-and micromotors for cancer therapy.

Carrier material	Drug	Target	Propulsion	Guidance	Release	Speed[µm/s]	Ref
*Structural units*							
plant-based microtubes	camptothecin	HeLa cells	magnetic	magnetic	drilling into cells, diffusion		[128]
*Loading units*							
red blood cells	DOX	-	ultrasound	magnetic	photothermal	50	
*Propulsion units*							
MC-1 magnetotactic bacteria	SN-38	HCT116 colorectal xenografts	bacterial flagella	magnetic aerotaxis	endocytosis of liposomes		[40]
*S. typhimurium* bacteria	paclitaxel	breast cancer cells	bacterial flagella	-	endocytosis of liposomes	3	[149]
*E. coli* bacteria	DOX	4Z T1 breast cancer cells	bacterial flagella	magnetic, chemotaxis	pH-dependent diffusion	10	[148]
*E. coli* bioadhesive bacteriabot	PMMA	mannose expressing cell line (HTB-9 cells)	bacterial flagella	-	adhesion to mannose-expressing cell surfaces		[182]
*E. coli* with red blood cells	DOX	-	bacterial flagella	magnetic	pH-dependent drug release from blood cells, NIR-triggered termination of bacteria	10	[147]
*S. typhimurium* NanoBEADS	-	tumor spheroids	bacterial flagella	bacteria translocation and proliferation	-	-	[150]
bovine sperm	DOX	tumor spheroids	sperm flagellum	magnetic	cell-cell-fusion	41	[145]
sea squirt sperm	DOX	ovarian cancer cells	sperm flagellum	chemotaxis	pH-induced death of sperm cells	200	[144]
human sperm	DOX	HeLa cells & human ovarian cancer tissue	sperm flagella	magnetic	cell-cell fusion	-	[187]
